# Influences of ski‐runs, meadow management and climate on the occupancy of reptiles and amphibians in a high‐altitude environment of Italy

**DOI:** 10.1002/ece3.11378

**Published:** 2024-05-20

**Authors:** Michele Chiacchio, Dennis Rödder, Klaus Henle, Annegret Grimm‐Seyfarth

**Affiliations:** ^1^ Department of Conservation Biology & Social‐Ecological Systems UFZ – Helmholtz Centre for Environmental Research Leipzig Germany; ^2^ Museum Koenig Bonn, Leibniz Institute for the Analysis of Biodiversity Change (LIB) Bonn Germany

**Keywords:** habitat change, mountains, multi‐season occupancy, *Rana temporaria*, *Vipera berus*, winter tourism, *Zootoca vivipara*

## Abstract

Alpine ecosystems harbour a rich and highly specialised biodiversity, which is particularly susceptible to anthropogenic disturbances such as habitat loss and fragmentation as well as to climate change. Combined with other forms of land‐use conversion, construction and maintenance of ski resorts can have severe consequences on alpine biodiversity. In this study, we show how one amphibian and two reptile species, namely *Rana temporaria*, *Zootoca vivipara* and *Vipera berus*, respond to such impacts by means of a multi‐season occupancy analysis. We found all three species both in and outside ski‐runs, showing that these habitats do not necessarily preclude their occurrence. Contrarily, this is influenced more by microhabitat availability, such as ground vegetation, humid areas and rock cover, rather than by macro‐characteristics like elevation or habitat type. Moreover, we found a climatic influence on the year‐to‐year occupancy change of the species, with activity‐month conditions being more relevant than overwintering ones. Our results demonstrate how, in the specific case of reptiles and amphibians, ski resorts do not necessarily limit species' occurrence and that a mild series of management actions might secure the species' persistence in the area.

## Graphical abstract

Multi‐season occupancy modelling was used to assess how high‐altitude herpetofauna respond to different climatic and environmental conditions. All three study species' occurrence was more influenced by microhabitat availability than by macro‐characteristics. Moreover, summer and spring temperatures influenced the year‐to‐year survivability of all three species.

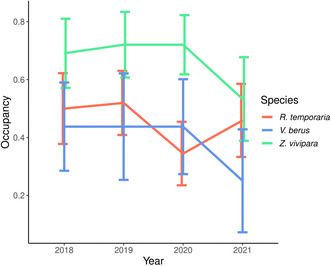

## 1 INTRODUCTION

Despite covering approximately only 3% of Europe's land surface (Nagy et al., 2003), mountain regions represent some of the areas with the richest biodiversity of the continent (Chemini & Rizzoli, 2003). Mountain ecosystems present rich and diversified communities adapted to unique environmental conditions, often characterised by endemism (Körner, 2004; Nagy & Grabherr, 2009; Viterbi et al., 2013), with even widespread, common species often showing strong behavioural, physiological and evolutionary differences from their lowland counterparts (Cheviron et al., 2013; Miaud et al., 1999; Roitberg & Smirina, 2006). Yet, like most ecosystems, mountain regions are threatened by the combined effect of land‐use conversion and climate change (Chemini & Rizzoli, 2003; Gehrig‐Fasel et al., 2007; Schmeller et al., 2022; Thuiller et al., 2006), with the shrinking of the suitable area of range‐restricted high‐altitude species (Dirnböck et al., 2011; Huang et al., 2013, 2014) and the expansion of forests, which is forecasted to grow up to 35% (Pellissier et al., 2013; Tasser et al., 2017), being the most relevant.

Because of their potentially extreme (local‐)adaptation, species inhabiting mountain areas are particularly susceptible to environmental changes (Viterbi et al., 2013). Even more so, this is true in the case of species with reduced dispersal abilities, such as reptiles and amphibians (Araújo et al., 2006; Deutsch et al., 2007; Grimm et al., 2014), for which relatively short distances are often insurmountable and even local extinction events might be irreversible due to the unlikely recolonisation from neighbouring populations (Ursenbacher et al., 2009).

Among the main causes of habitat alteration in mountain areas, ski resort‐based tourism represents a major one, yet often understudied (Caprio et al., 2011; Sato, Wood, Schroeder, Michalel, et al., 2014). Its implementation has repercussions both above and below the timberline (Caprio et al., 2011; Negro et al., 2009; Rixen & Rolando, 2013; Sachot et al., 2003; Thiel et al., 2008). Such impacts begin during the ski‐runs construction in the form of clear‐cutting, machine grading, removal of boulders and logs, and artificial seeding (Negro et al., 2009; Rixen & Rolando, 2013). Impacts continue afterwards with ski‐run maintenance through summer mowing, ground levelling and winter snow‐grooming, which reduces heat loss and gas exchange of the soil (Negro et al., 2010; Rixen & Rolando, 2013). Moreover, the use of artificial snow induces a delay in snowmelt as well as soil warming, shifting the flowering time of plants with cascading effects on the higher trophic levels (Rixen et al., 2004). It might also add pollutants and chemicals to soils and waters altering the fragile nutrient balance (Rixen et al., 2003). However, while the effects of ski resorts on plants and arthropods are comparably well known (Negro et al., 2013; Wipf et al., 2005), studies on their impacts on other species, such as amphibians and reptiles, remain largely unknown (Sato, Wood, & Lindenmayer, 2013).

Amphibians and reptiles are particularly threatened worldwide (Böhm et al., 2013; Gibbons et al., 2000; Stuart et al., 2004). Despite large extinctions of amphibians and reptiles in high‐altitude environments having been predicted both as a direct and indirect consequence of climate change and habitat fragmentation (Huang et al., 2013; McCain & Colwell, 2011; Sheldon et al., 2011), herpetofauna in mountain habitats from temperate regions is still very little studied (Corn, 2005; Slatyer et al., 2021; Tan et al., 2023). Nevertheless, amphibians and reptiles in these areas represent an important part of trophic chains (Sato, Wood, Schroeder, Michalel, et al., 2014; Sergio et al., 2002; Sztatecsny et al., 2013) as well as a highly diversified gene pool, with even widely distributed species presenting large differences from neighbouring conspecific populations (Savage et al., 2010; Ursenbacher et al., 2009).

Amphibians and reptiles have different environmental requirements than other species (Buckley et al., 2012; Sheldon et al., 2011); hence, it is possible that they will not respond identically to other animals to such forms of landscape alteration. For instance, the clear‐keeping of ski‐runs could actually be beneficial for ectothermic species that might take advantage from the increased solar radiation (Huang et al., 2013, 2014). On the other hand, the depletion of prey sources (Negro et al., 2009, 2010), the removal of suitable refuges (Amo et al., 2007; Sato, Schroder, Green, Michael, et al., 2014; Sato, Wood, Schroeder, et al., 2013; Sato, Wood, Schroeder, Green, et al., 2014; Sato, Wood, Schroeder, Michalel, et al., 2014) and the homogenising of microclimates along ski‐runs (Sato, Schroder, Green, Michael, et al., 2014, Sato, Wood, Schroeder, Green, et al., 2014, Sato, Wood, Schroeder, Michalel, et al., 2014) might lead to an unsuitable environment to reduced individuals' health status and time available for activity.

In this study, we used detection data from 2018 to 2021 for three not‐alpine specialist but widespread species, namely *Rana temporaria*, *Zootoca vivipara* and *Vipera berus*, to examine reptile and amphibian occupancy in high‐altitude environments in relation to anthropogenic and environmental factors. Although all three species are some of the most widespread Eurasian ectotherms, the increasing acknowledgement on the ecological relevance of common species pushes for including also these in monitoring programmes and conservation studies (Gaston, 2008). Moreover, although in most of their range, the species are found along the whole elevational gradient, in the Alps, which represent the southern border of their distributions, they are mostly confined above 1600 m a.s.l. (Di Cerbo & Sassi, 2023). Therefore, we consider them as suitable study species for this ecological study as well as an interesting target considering the different response to ecological constraints between reptiles and amphibians at the southern margins of their distribution (Cunningham et al., 2016).

We predicted that the three species will avoid ski resorts and will be found more consistently in pastural and alpine meadows. Furthermore, we expected the availability of microhabitats to be the second most relevant criteria for the occupancy of the species while we predicted climate and meteorological conditions to have the main role in determining year to year occupancy changes.

## 2 METHODS

### 2.1 Study site

**FIGURE 1:**
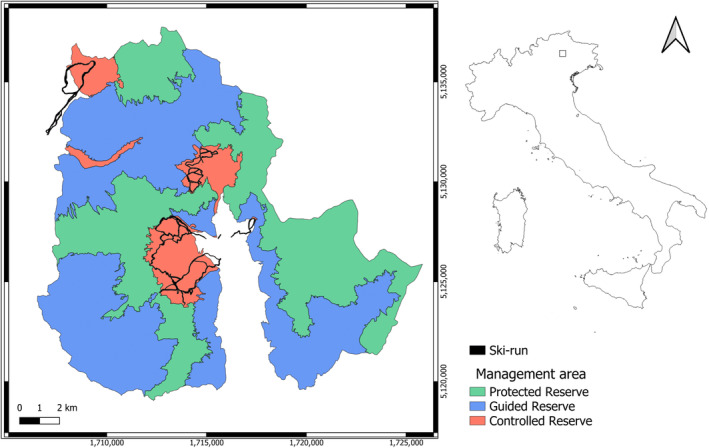
Paneveggio‐Pale di San Martino Nature Park and its internal divisions.

The study was conducted in Paneveggio‐Pale di San Martino Nature Park (46°17.357′ N, 011°46.116′ E), in Trentino‐Alto Adige Region, Italy (Figure 1). The park is divided into three management areas: a core area (Protected Reserve), a buffer zone (Guided Reserve) where traditional forms of management like extensive grazing are allowed, and anthropogenic areas (Controlled Reserve) in which most of the infrastructures, villages and two ski resorts, namely San Martino di Castrozza (9.85 km^2^, 60 km of ski‐runs: 6.09 km of ski‐runs per km^2^) and Passo Rolle (5.33 km^2^, 15 km of ski‐runs: 2.81 km of ski‐run per km^2^) are localised. We conducted our data collection in the north‐eastern area of the park (Passo Rolle and adjacent areas). The area spans from 1890 to 2100 m a.s.l. and consists of open habitats dominated by grass and shrub vegetation, alternating with coniferous forests of Norway spruce (*Picea abies*) and European larch (*Larix decidua*).

For the purpose of the study, we initially defined four different habitat types: ski‐runs, disturbed meadows (grasslands neighbouring ski‐runs or roads), traditional meadows (anthropogenically deforested areas used for grazing) and alpine meadows (natural grasslands above the timberline). The first two types were found in the Controlled Reserve; the other two in the Guided and in the Protected Reserve, respectively. Despite this classification, however, a clear distinction between habitat types (with the exception of ski‐runs) was missing, and most of the environmental characteristics were plot‐ rather than habitat‐specific. Grazers, for instance, were present in most of the plots and not just on those found in the pastural meadows as expected, whereas vegetation height was more different among single plots than among habitats. Therefore, this classification is meant mostly to acknowledge parameters that we did not measure directly but that still might be present, such as pollutants resulting from artificial snow usage.

### 2.2 Monitoring surveys

During each of the four years (2018–2021), we surveyed a total of 16 plots evenly distributed among the four habitat types. For logistic reasons, in 2021, we did not survey the four plots found in alpine meadows.

**FIGURE 2:**
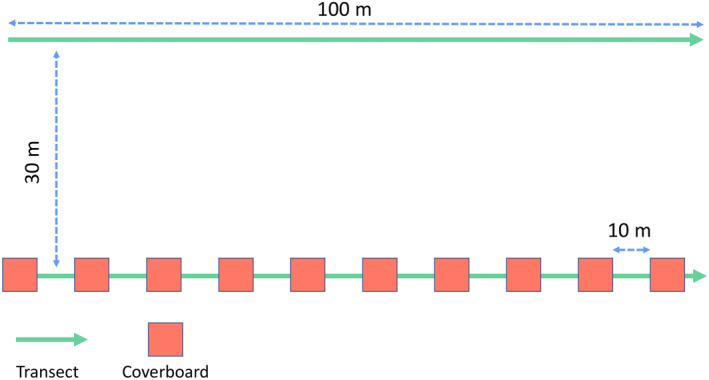
Schematic representation of the plots' setup.

Each plot consisted of two parallel 100‐m transects spaced circa 30 m apart, with each transect further subdivided into 10 m intervals called ‘cells’. We employed a combination of visual encounter surveys (VES) and coverboards (40 × 50 cm, corrugated bitumen, ≃0.5 kg), with one coverboard in every cell of one of the two transects (Figure 2). In all four years, surveys were conducted by the same observer who walked the transects at a standard pace using as much time as was needed to examine the area thoroughly and to lift the coverboards (approximately 50 min). We conducted the surveys every morning between 8:00 and 13:00 from June to August.

Every year (‘primary periods’), each plot was surveyed for nine consecutive days (‘secondary periods’) after an initial day for placing the coverboards on the ground, and another day to let them settle. We surveyed plots by group of two or three per day, depending on logistics and position. Following the nine days of sampling, the coverboards were removed and placed in another group of plots. Survey order was rotated both daily and yearly to ensure a complete temporal coverage and to minimise seasonal effects on sampling.

### 2.3 Environmental parameters

We collected a combination of constant, yearly and daily environmental variables (Table 1). Specifically, constant variables did not change over the four years of survey while yearly variables could change among years but not within them. Finally, daily variables were only relevant for the survey conducted during that day.

**TABLE 1:** Variables used in the modelling of multi‐season occupancy of *Rana temporaria*, *Zootoca vivipara* and *Vipera berus* in Paneveggio‐Pale di San Martino Nature Park, Trentino (IT), 2018–2021.

Probability of	Parameter name (abbreviation used in Tables 3, 4, 5)	Scale	Range
Detection	Year (‘year’)	Yearly	2018–2021
	Julian date (‘date’)	Daily	168–234
	Days since last rain (‘rain’)	Daily	0–8
	Air temperature (‘air’)	Daily	2.91–22.64°C
	Ground temperature^a^	Daily	4.68–29.46°C
	Weather conditions^a^	Daily	“sun”, “cloud”, “overcast”, “rain”, “fog”
	Wind intensity^a^	Daily	“no”, “low”, “strong”
	Survey hour (‘hour’)	Daily	“early morning”, “mid‐morning”, “late morning”
	Grazers (‘cow’)	Daily	“no”, “yes”
	Rock density (‘rock’)	Constant	0%–85%
	Bush density (‘bush’)	Yearly	0%–85%
	Log density (‘log’)	Yearly	0%–40%
	Vegetation height (‘veg’)	Yearly	5.4–23.5 cm
Initial occupancy	Habitat (‘habitat’)	Constant	“ski‐run”, “disturbed meadow”, “pastural meadow”, “alpine meadow”
	Elevation^a^	Constant	1890–2111 m a.s.l.
	Water distance (‘water’)	Constant	53–709 m
	Ecotone distance (‘ecotone’)	Constant	18–529 m
	Ski‐lift distance^a^	Constant	0–2530 m
	Tree density^a^	Constant	0%–65%
	Orientation	Constant	“N‐S”, “S‐N”, “E‐W”
	Slope	Constant	“parallel”, “perpendicular”
	Humidity density (‘humid’)	Constant	0%–25%
	Rock density (‘rock’)	Constant	0%–85%
	Bush density (‘bush’)	Yearly	0%–85% (1st year only)
	Log density (‘log’)	Yearly	0%–30% (1st year only)
	Vegetation height (‘veg’)	Yearly	10.25%–22.75% (1st year only)
Colonisation and Extinction	Year (‘year’)	Yearly	2018–2021
	Spring precipitations (‘sprr’)	Yearly	445.4–577 cm
	Summer precipitations (‘sumr’)	Yearly	261.4–481.4 cm
	Winter temperatures (‘wint’)	Yearly	−1.8 to −0.22°C
	Bush density (‘bush’)	Yearly	0%–85%
	Log density (‘log’)	Yearly	0%–40%
	Vegetation height (‘veg’)	Yearly	5.4–23.5 cm

^a^ Variables discarded because of collinearity.

As constant variables, we measured the elevation at the centre of the plot, the shortest distance from the ecotone, from ski‐lifts and from a permanent standing water body. We also adopted two categorical variables to describe the plot orientation and whether it was perpendicular or parallel to the elevational gradient, plus the presence/absence of rocks and humid spots, such as brooks, bogs and seeps, for every cell. These last two variables were then upscaled for the whole plot by calculating the percentage of cells in which they were present as a proxy for their density in the plots. For instance, a rock presence/absence history of 1,001,000,001, indicating that there were rocks in the first, fourth and last cell of a transect, resulting in a rock density of 30% for that transect.

Yearly variables included the presence/absence of bushes and logs, as well as the approximate mean ground‐vegetation height. Unlike rocks, these were more prone to annual fluctuations both because of human influences and natural environmental conditions. Again, these values were upscaled for the whole plot as shown for the constant variable ‘rock density’ while the vegetation height was upscaled by averaging the different cell values. We also gathered long‐term weather data from the closest weather station available (station number T0103, www.meteotrentino.it). Because our surveys started in June, we used the total precipitation from the previous spring (May–July) and from the previous summer (August–September) plus the average temperatures from the recently terminated winter (October–April). These are considered as the most important climatic parameters shaping population dynamics in both amphibian and reptile species (Le Galliard et al., 2010; Pikulik et al., 2001) as they determine the number of juveniles recruited during the last breeding season and the animal conditions before entering hibernation (Reading & Clarke, 1995) as well as the overwinter survival probability (Muths et al., 2020).

Daily variables were plot‐specific rather than cell‐specific as they remained constant for the time of the survey and were collected before the beginning of the survey. These included the Julian date, the survey hour (‘early’: 8.00–9.30, ‘mid‐morning’: 9.30–11.00, ‘late’: 11.00–12.30), the number of days since the last rain, the intensity of wind, the presence of grazers and the weather conditions. While inspecting the transects, we also measured air and ground temperature every 10 m using a probe thermometer. These were then averaged for the whole plot.

### 2.4 Occupancy analyses

We performed all our analyses with R 4.0.3 (R Core Team, 2020). We analysed presence/absence data of reptiles and amphibians using multi‐season occupancy models (MacKenzie et al., 2003) as implemented by the R‐package *unmarked* (Chandler et al., 2020). Occupancy analyses rely on presence/absence data from different sampling units to estimate detection probability (*p*) and occupancy (*Ψ*) for the different years, assuming no demographical changes within the primary periods. The multi‐season model allows to derive also colonisation (*γ*) and extinction rates (*ε*), measuring the changes in occupancy between the primary periods. The daily detection histories were constructed using the plots as sampling units, with ‘1’ marking a positive encounter with the subject species and ‘0’ indicating the animal was not observed.

Firstly, we checked for collinearity between variables using Spearman's rank correlation test for continuous covariates, chi‐square test for categorical ones, and Kruskal–Wallis test between the two kinds. If parameters were correlated (the threshold was set at *r* > |.6| for correlation and 5% for significance), we retained the one that likely was biologically more relevant. Elevation, distance from ski‐lifts and tree cover were correlated with most of the other variables and thus discarded. Among daily variables, weather conditions, ground temperatures and wind intensity showed strong correlations with most of the other variables, and therefore discarded. All retained measures were *z*‐transformed to a mean of 0 and a standard deviation of 1.

As observatory covariates, we used all daily variables as well as vegetation height, and the density of bushes, logs and rocks, as we assumed a stronger hiding capacity in shelter‐rich habitats (Table 1). As site covariates, we used all constant variables plus vegetation height, bush and log density. Finally, as yearly covariates, we used all the long‐term weather data, bush and log density, and vegetation height.

Model selection was performed using the R‐package *MuMIn* (Barton, 2020). We adopted a four‐step procedure to build the model. Firstly, we selected those variables influencing detection probability while holding all the remaining parameters constant. Then, we added variables influencing occupancy to the first ranked detection model. Finally, we used the first ranked detection/occupancy model and added variables predicting colonisation and extinction rates. In the different steps, models were ranked using Aikake's Information Criterion (AIC; Burnham & Anderson, 2002) and we selected the best combination of covariates choosing the model with the lowest AIC value. Models with ΔAIC < 2 were considered to be equivalent (Burnham & Anderson, 2002). We then summed the AIC weights (ωAIC) of each covariate across models to define their importance.

## 3 RESULTS

In total, we conducted 189 days of surveys. The species most frequently observed were *Zootoca vivipara*, having been seen 159 times and showing a naïve occupancy of 0.75 (i.e. the proportion of sites with positive detection across all years), and *Rana temporaria*, which was observed in 126 different occasions (naïve occupancy = 0.62). These species were observed in all four habitat types (Table 2). *Vipera berus*, on the other hand, was never observed in the pastural meadow and was only seen 61 times (naïve occupancy = 0.31).

**TABLE 2:** Total number of yearly encounters for each species in the different habitat types.

Species	Habitat	Year			
		2018	2019	2020	2021
*Rana temporaria*	Ski‐run	7	11	8	7
	Disturbed meadow	13	5	5	3
	Pastural meadow	24	18	9	15
	Alpine meadow	0	1	0	NA
*Zootoca vivipara*	Ski‐run	10	7	11	10
	Disturbed meadow	21	16	15	13
	Pastural meadow	10	18	13	6
	Alpine meadow	0	3	6	NA
*Vipera berus*	Ski‐run	1	7	11	3
	Disturbed meadow	8	10	9	10
	Pastural meadow	0	0	0	0
	Alpine meadow	0	1	1	NA

*Note*: NA: not available because habitat was not surveyed.

All three species were observed more frequently during warmer days and air temperature was always the most significant parameter predicting detectability (*ω*_*i*_ = 1.00 for all). While this was the only parameter whose importance was higher than 0.50 for *R*. *temporaria* (Table 3), both *Z*. *vivipara* and *V*. *berus* detectability was also driven by a wide range of environmental parameters (Tables 4 and 5), including bush density (*ω*_*i*_ = 0.64 and 0.99, respectively), fallen logs (*ω*_*i*_ = 0.88 and 0.97) and, more importantly, rock availability (*ω*_*i*_ = 0.92 and 1.00). All these microhabitats positively influenced the species detectability except for rocks for *Vipera berus*, which decreased its detectability. *Z*. *vivpara*'s detectability was also predicted by daily variables like the Julian date (*ω*_*i*_ = 0.97) and the number of days since the last rain (*ω*_*i*_ = 0.63), which had a negative effect, and the hour of survey (*ω*_*i*_ = 0.59), which determined a higher observation rate during the central hours of the morning and decreased it towards the extremes.

**TABLE 3:** Model selection (ΔAIC < 2.00) of initial occupancy (*Ψ*), detection probability (*p*), colonisation (*γ*) and extinction (*ε*) of *Rana temporaria*.

Step	Model	AIC	ΔAIC	*ω*_*i*_
1	*Ψ*(.) *γ*(.) *ε*(.) *p*(air)	396.0	0.00	0.231
	*Ψ*(.) *γ*(.) *ε*(.) *p*(air + hour)	396.9	0.85	0.151
	*Ψ*(.) *γ*(.) *ε*(.) *p*(air + rock)	397.2	1.19	0.127
	*Ψ*(.) *γ*(.) *ε*(.) *p*(air + log)	397.6	1.54	0.107
	*Ψ*(.) *γ*(.) *ε*(.) *p*(air + date)	397.6	1.54	0.107
	*Ψ*(.) *γ*(.) *ε*(.) *p*(air + rain)	397.7	1.62	0.102
	*Ψ*(.) *γ*(.) *ε*(.) *p*(air + cow)	397.9	1.91	0.089
	*Ψ*(.) *γ*(.) *ε*(.) *p*(air + bush)	398.0	1.97	0.086
2	*Ψ*(humid + bush) *γ*(.) *ε*(.) *p*(air)	380.8	0.00	0.151
	*Ψ*(humid + bush + slope) *γ*(.) *ε*(.) *p*(air)	380.9	0.05	0.148
	*Ψ*(humid + slope + ecotone) *γ*(.) *ε*(.) *p*(air)	380.9	0.06	0.147
	*Ψ*(humid +bush + habitat) *γ*(.) *ε*(.) *p*(air)	380.9	0.08	0.145
	*Ψ*(humid + slope + veg) *γ*(.) *ε*(.) *p*(air)	381.2	0.36	0.126
	*Ψ*(humid + bush + log) *γ*(.) *ε*(.) *p*(air)	382.8	1.95	0.057
	*Ψ*(humid + bush + rock) *γ*(.) *ε*(.) *p*(air)	382.8	1.96	0.057
	*Ψ*(humid + bush + ecotone) *γ*(.) *ε*(.) *p*(air)	382.8	1.96	0.057
	*Ψ*(humid + bush + veg) *γ*(.) *ε*(.) *p*(air)	382.8	1.97	0.057
	*Ψ*(humid + bush + water) *γ*(.) *ε*(.) *p*(air)	382.8	1.97	0.056
3	*Ψ*(humid + bush) *γ*(veg + bush) *ε*(.) *p*(air)	374.2	0.00	0.307
	*Ψ*(humid + bush) *γ*(veg) *ε*(.) *p*(air)	374.8	0.54	0.234
	*Ψ*(humid + bush) *γ*(veg + sumr) *ε*(.) *p*(air)	375.4	1.16	0.172
	*Ψ*(humid + bush) *γ*(veg + bush + sumr) *ε*(.) *p*(air)	375.4	1.16	0.172
	*Ψ*(humid + bush) *γ*(veg + bush + log) *ε*(.) *p*(air)	376.2	1.98	0.114
4	*Ψ*(humid + bush) *γ*(veg + bush) *ε*(sumr) *p*(air)	373.2	0.00	0.178
	*Ψ*(humid + bush) *γ*(veg + bush) *ε*(.) *p*(air)	374.2	1.04	0.106
	*Ψ*(humid + bush) *γ*(veg + bush) *ε*(sprr + wint) *p*(air)	374.4	1.19	0.098
	*Ψ*(humid + bush) *γ*(veg + bush) *ε*(year) *p*(air)	374.4	1.20	0.098
	*Ψ*(humid + bush) *γ*(veg + bush) *ε*(sumr + sprr) *p*(air)	374.4	1.20	0.098
	*Ψ*(humid + bush) *γ*(veg + bush) *ε*(sumr + wint) *p*(air)	374.4	1.21	0.097
	*Ψ*(humid + bush) *γ*(veg + bush) *ε*(sumr + bush) *p*(air)	374.5	1.30	0.093
	*Ψ*(humid + bush) *γ*(veg + bush) *ε*(sumr + veg) *p*(air)	374.5	1.33	0.091
	*Ψ*(humid + bush) *γ*(veg + bush) *ε*(sumr + log) *p*(air)	375.0	1.78	0.073
	*Ψ*(humid + bush) *γ*(veg + bush) *ε*(sumr + bush + veg) *p*(air)	375.1	1.94	0.067

*Note*: ΔAIC is the difference from the lowest AIC value; *ω*_*i*_ is the AIC model weight.

**TABLE 4:** Model selection (ΔAIC < 2.00) of initial occupancy (*Ψ*), detection probability (*p*), colonisation (*γ*) and extinction (*ε*) of *Zootoca vivipara*.

Step	Model	AIC	ΔAIC	*ω*_*i*_
1	*Ψ*(.) *γ*(.) *ε*(.) *p*(air + date + rock + log + bush + rain + hour)	480.2	0.00	0.165
	*Ψ*(.) *γ*(.) *ε*(.) *p*(air + date + rock + log + bush + rain)	480.6	0.42	0.134
	*Ψ*(.) *γ*(.) *ε*(.) *p*(air + date + rock + log + bush + hour)	480.9	0.76	0.113
	*Ψ*(.) *γ*(.) *ε*(.) *p*(air + date + rock + log + rain + hour)	481.3	1.16	0.092
	*Ψ*(.) *γ*(.) *ε*(.) *p*(air + date + rock + log + bush)	481.6	1.42	0.081
	*Ψ*(.) *γ*(.) *ε*(.) *p*(air + date + rock + log + bush + rain + hour + year)	481.6	1.43	0.081
	*Ψ*(.) *γ*(.) *ε*(.) *p*(air + date + rock + log + bush + rain + hour + cow)	481.8	1.63	0.073
	*Ψ*(.) *γ*(.) *ε*(.) *p*(air + date + rock + log + hour)	481.9	1.77	0.068
	*Ψ*(.) *γ*(.) *ε*(.) *p*(air + date + rock + log + bush + rain + year)	482.0	1.82	0.066
	*Ψ*(.) *γ*(.) *ε*(.) *p*(air + date + rock + log + rain)	482.0	1.85	0.065
	*Ψ*(.) *γ*(.) *ε*(.) *p*(air + date + rock + log + bush + rain + hour + veg)	482.1	1.96	0.062
2^a^	*Ψ*(water + ecotone) *γ*(.) *ε*(.) *p*(air + date + rock + log + bush + rain + hour)	466.4	0.00	0.288
	*Ψ*(humid + bush) *γ*(.) *ε*(.) *p*(air + date + rock + log + bush + rain + hour)	466.5	0.02	0.285
	*Ψ*(water + ecotone + veg) *γ*(.) *ε*(.) *p*(air + date + rock + log + bush + rain + hour)	468.4	1.98	0.107
	*Ψ*(water + bush + ecotone) *γ*(.) *ε*(.) *p*(air + date + rock + log + bush + rain + hour)	468.4	1.98	0.107
	*Ψ*(water + humid + bush) *γ*(.) *ε*(.) *p*(air + date + rock + log + bush + rain + hour)	468.4	1.99	0.107
	*Ψ*(water + ecotone + slope) *γ*(.) *ε*(.) *p*(air + date + rock + log + bush + rain + hour)	468.4	1.99	0.17
3	*Ψ*(humid + bush) *γ*(veg) *ε*(.) *p*(air + date + rock + log + bush + rain + hour)	466.4	0.00	0.371
	*Ψ*(humid + bush) *γ*(.) *ε*(.) *p*(air + date + rock + log + bush + rain + hour)	466.5	0.10	0.352
	*Ψ*(humid + bush) *γ*(veg + log) *ε*(.) *p*(air + date + rock + log + bush + rain + hour)	468.3	1.96	0.139
	*Ψ*(humid + bush) *γ*(veg + wint) *ε*(.) *p*(air + date + rock + log + bush + rain + hour)	468.3	1.99	0.137
4	*Ψ*(humid + bush) *γ*(veg) *ε*(bush + log + sprr) *p*(air + date + rock + log + bush + rain + hour)	461.7	0.00	0.334
	*Ψ*(humid + bush) *γ*(veg) *ε*(bush + log + sumr) *p*(air + date + rock + log + bush + rain + hour)	461.9	0.19	0.305
	*Ψ*(humid + bush) *γ*(veg) *ε*(bush + wint + veg) *p*(air + date + rock + log + bush + rain + hour)	462.9	1.23	0.181
	*Ψ*(humid + bush) *γ*(veg) *ε*(bush + log + wint) *p*(air + date + rock + log + bush + rain + hour)	462.9	1.24	0.180

*Note*: ΔAIC is the difference from the lowest AIC value; *ω*_*i*_ is the AIC model weight.

^a^ The second model was maintained as best model as presence of humid areas and bush density was considered as more relevant for the species.

**TABLE 5:** Model selection (ΔAIC <2.00) of initial occupancy (*Ψ*), detection probability (*p*), colonisation (*γ*) and extinction (*ε*) of *Vipera berus*.

Step	Model	AIC	ΔAIC	*ω*_*i*_
1	*Ψ*(.) *γ*(.) *ε*(.) *p*(air + rock + year + bush + log + veg)	146.9	0.00	0.388
	*Ψ*(.) *γ*(.) *ε*(.) *p*(air + rock + year + bush + log + veg + hour)	148.6	1.67	0.168
	*Ψ*(.) *γ*(.) *ε*(.) *p*(air + rock + year + bush + log + veg + date)	148.8	1.86	0.153
	*Ψ*(.) *γ*(.) *ε*(.) *p*(air + rock + year + bush + log + veg + rain)	148.9	1.94	0.147
	*Ψ*(.) *γ*(.) *ε*(.) *p*(air + rock + year + bush + log + veg + cow)	148.9	1.98	0.144
2^a^	*Ψ*(rock + ecotone + water) *γ*(.) *ε*(.) *p*(air + rock + year + bush + log + veg)	134.8	0.00	0.147
	*Ψ*(rock + ecotone + log) *γ*(.) *ε*(.) *p*(air + rock + year + bush + log + veg)	134.8	0.01	0.146
	*Ψ*(rock + orientation) *γ*(.) *ε*(.) *p*(air + rock + year + bush + log + veg)	134.8	0.04	0.144
	*Ψ*(rock + log + humid) *γ*(.) *ε*(.) *p*(air + rock + year + bush + log + veg)	135.0	0.25	0.130
	*Ψ*(ecotone + slope + veg) *γ*(.) *ε*(.) *p*(air + rock + year + bush + log + veg)	135.2	0.42	0.119
	*Ψ*(rock + log + veg) *γ*(.) *ε*(.) *p*(air + rock + year + bush + log + veg)	135.2	0.42	0.119
	*Ψ*(rock + log + water) *γ*(.) *ε*(.) *p*(air + rock + year + bush + log + veg)	135.9	1.08	0.086
	*Ψ*(rock + ecotone + log + water) *γ*(.) *ε*(.) *p*(air + rock + year + bush + log + veg)	136.8	1.98	0.055
	*Ψ*(rock + ecotone + water + veg) *γ*(.) *ε*(.) *p*(air + rock + year + bush + log + veg)	136.8	1.98	0.055
3	*Ψ*(rock + ecotone + log) *γ*(.) *ε*(.) *p*(air + rock + year + bush + log + veg)	134.8	0.00	1.000
4	*Ψ*(rock + ecotone + log) *γ*(.) *ε*(wint) *p*(air + rock + year + bush + log + veg)	134.1	0.00	0.087
	*Ψ*(rock + ecotone + log) *γ*(.) *ε*(sprr) *p*(air + rock + year + bush + log + veg)	134.1	0.00	0.087
	*Ψ*(rock + ecotone + log) *γ*(.) *ε*(sumr) *p*(air + rock + year + bush + log + veg)	134.1	0.01	0.087
	*Ψ*(rock + ecotone + log) *γ*(.) *ε*(bush + veg + sprr) *p*(air + rock + year + bush + log + veg)	134.1	0.01	0.087
	*Ψ*(rock + ecotone + log) *γ*(.) *ε*(bush + veg + wint) *p*(air + rock + year + bush + log + veg)	134.4	0.30	0.075
	*Ψ*(rock + ecotone + log) *γ*(.) *ε*(bush + veg + sumr) *p*(air + rock + year + bush + log + veg)	134.5	0.33	0.074
	*Ψ*(rock + ecotone + water) *γ*(.) *ε*(bush + sprr) *p*(air + rock + year + bush + log + veg)	134.6	0.44	0.070
	*Ψ*(rock + ecotone + water) *γ*(.) *ε*(bush + wint) *p*(air + rock + year + bush + log + veg)	134.7	0.59	0.065
	*Ψ*(rock + ecotone + water) *γ*(.) *ε*(bush + sumr) *p*(air + rock + year + bush + log + veg)	134.7	0.59	0.065
	*Ψ*(rock + ecotone + water) *γ*(.) *ε*(.) *p*(air + rock + year + bush + log + veg)	134.8	0.69	0.062
	*Ψ*(rock + ecotone + water) *γ*(.) *ε*(log + sprr) *p*(air + rock + year + bush + log + veg)	135.4	1.32	0.045
	*Ψ*(rock + ecotone + water) *γ*(.) *ε*(log + wint) *p*(air + rock + year + bush + log + veg)	135.4	1.33	0.045
	*Ψ*(rock + ecotone + water) *γ*(.) *ε*(log + sumr) *p*(air + rock + year + bush + log + veg)	135.4	1.33	0.045
	*Ψ*(rock + ecotone + water) *γ*(.) *ε*(veg + sprr) *p*(air + rock + year + bush + log + veg)	135.9	1.75	0.036
	*Ψ*(rock + ecotone + water) *γ*(.) *ε*(veg + wint) *p*(air + rock + year + bush + log + veg)	135.9	1.76	0.036
	*Ψ*(rock + ecotone + water) *γ*(.) *ε*(veg + sumr) *p*(air + rock + year + bush + log + veg)	135.9	1.76	0.036

*Note*: ΔAIC is the difference from the lowest AIC value; *ω*_*i*_ is the AIC model weight.

^a^ The second model was maintained as best model as presence of humid areas and bush density was considered as more relevant for the species.

The occupancy of *Z*. *vivipara* decreased with increasing distance from water (*ω*_*i*_ = 0.57) and from the ecotone (*ω*_*i*_ = 0.43), but a model including the presence of humid spots (*ω*_*i*_ = 0.54) and bush density (*ω*_*i*_ = 0.46) was equally supported (ΔAIC = 0.02; Table 4). Similarly, also *Rana temporaria* was mostly found in plots that were rich in humid spots (*ω*_*i*_ = 1.00) and with high density of bush cover (*ω*_*i*_ = 0.44). For this species, several other parameters appeared in models with comparable AIC values, although only the plot's slope had an importance of 0.50 (Table 3). *Vipera berus* on the other hand showed a more distinct variable selection and its occupancy was positively determined by rock availability (*ω*_*i*_ = 0.78). Conversely, distance from the ecotone (*ω*_*i*_ = 0.51) and from a water source (*ω*_*i*_ = 0.41) had an opposite effect. A second model (ΔAIC = 0.01) included log density (*ω*_*i*_ = 0.41) instead of water distance.

**FIGURE 3:**
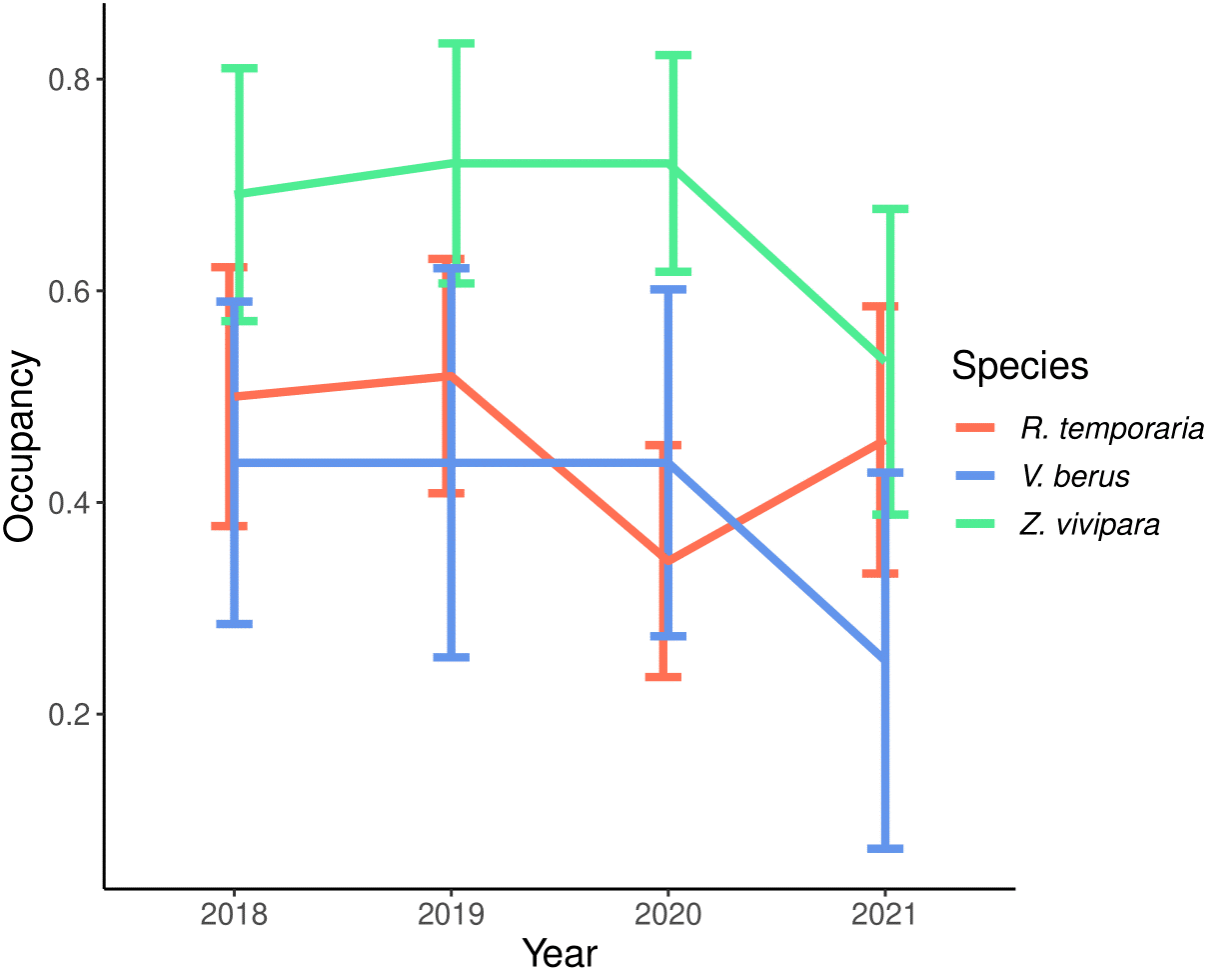
Occupancy values with standard errors for the three species sampled: *Rana temporaria*, *Zootoca vivipara* and *Vipera berus*.

For the two reptile species, occupancy remained constant during the first three years of survey and, in the colonisation model selection, the null models appeared to be the best model for both species although a model, including vegetation height was equally supported for *Zootoca vivipara* (ΔAIC = 0.10; Table 4). However, both species showed a strong drop in their occupancy in 2021 (Figure 3), with bush density being the most important predictor (*ω*_*i*_ = 0.88 for *Z*. *vivipara*, and 0.50 for *V*. *berus*), followed by log density for *Z*. *vivipara* (*ω*_*i*_ = 0.73) and vegetation height for *V*. *berus* (*ω*_*i*_ = 0.41). Although less important, there was also a climatic influence for both species, specifically spring precipitation appeared in the best model for *Z*. *vivipara* (*ω*_*i*_ = 0.37) and all three weather covariates (spring precipitations, *ω*_*i*_ = 0.39; winter temperatures, *ω*_*i*_ = 0.38; summer precipitations, *ω*_*i*_ = 0.38) appeared in the three top‐selected models regarding the extinction rate of *V*. *berus* (Table 5).

**FIGURE 4:**
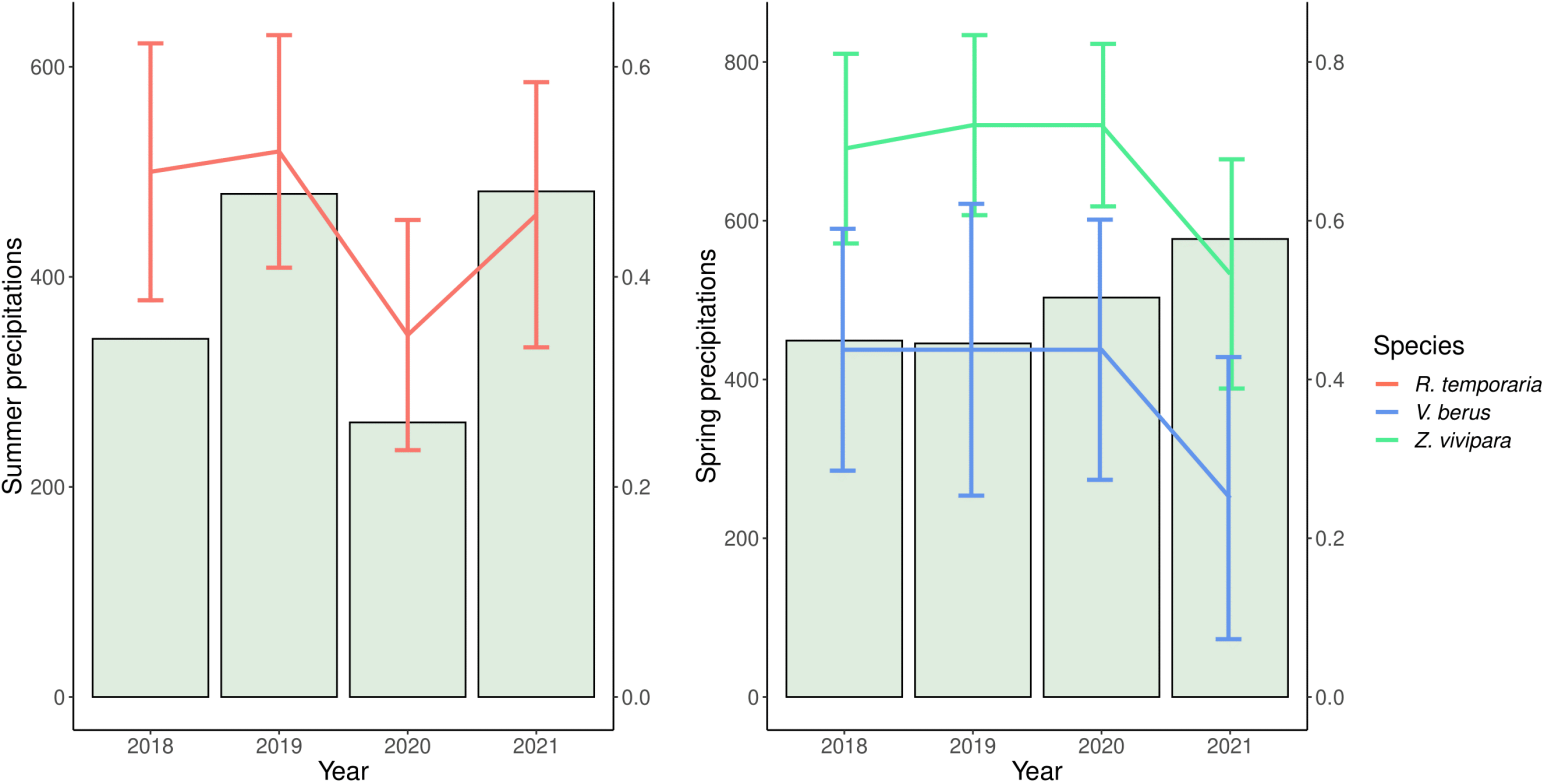
Correlation between species occupancy and previous summer precipitations (left) and previous spring precipitations (right).

On the other hand, the occupancy of *R*. *temporaria* fluctuated strongly during the survey years, dropping from 0.51 in 2019 to 0.34 in 2020 and then recovering back to 0.45 in 2021 (nearly as it was in 2018; Figure 3). Surges in colonisation probabilities were mostly due to changes in vegetation height (*ω*_*i*_ = 0.84) and bush density (*ω*_*i*_ = 0.57), with both parameters' increase leading to a higher presence probability (Table 3). Conversely, disappearance resulted to be mostly caused by climatic parameters, namely by summer precipitations (*ω*_*i*_ = 0.50; Table 3), which was strongly correlated with the following year's occupancy (Figure 4). However, the null model resulted to be equally supported (ΔAIC = 1.04).

## 4 DISCUSSION

With this study, we provide an ecological assessment of reptiles and amphibians in high‐altitude environments in the strongly anthropised landscape of the European Alps, with a particular focus on the effects of ski resorts and related infrastructure. Previous studies on the effects of winter tourism on reptiles' ecology and conservation had been conducted mostly in the Australian highlands (Sato, Schroder, Green, Michael, et al., 2014; Sato, Wood, Schroeder, et al., 2013; Sato, Wood, Schroeder, Green, et al., 2014; Sato, Wood, Schroeder, Michalel, et al., 2014; Shine et al., 2002), with only one project implemented in Europe, specifically in the Pyrenees (Amo et al., 2007). Moreover, no study had addressed the same issue from the amphibian perspective (Sato, Wood, & Lindenmayer, 2013).

Despite starting from quite different initial occupancy values, our results showed that the two reptile species, namely *Zootoca vivipara* and *Vipera berus*, presented a very similar pattern throughout the study period while the one of *Rana temporaria* followed a different trend and its occupancy fluctuated more intensively from year to year (Figure 3). Their wide distribution throughout the area is in line with the generalist behaviour at least for *Rana temporaria* and *Zootoca vivipara*, which are known to occur in a wide range of habitats and to tolerate a certain level of disturbance (Miaud et al., 1999; Surget‐Groba et al., 2001) as well as to show strong demographic fluctuations from year to year in the case of *R*. *temporaria* (Chiacchio et al., 2022; Loman & Anderson, 2007). *Vipera berus* on the other hand is more susceptible to environmental change and human disturbance (Nöllert, 2004; Reading et al., 1996), explaining its lower occupancy values. However, unlike many specialist species for which ski resorts are always an inaccessible environment (Sato, Wood, Schroeder, et al., 2013), all three species were found on ski‐runs as well as far from them, with micro‐environmental parameters, such as vegetation height and microhabitat density, being generally more relevant than macro‐characteristics, like habitat type or elevation.

Overall, our results confirm the strong dependence of *R*. *temporaria* and *Z*. *vivipara* on humid areas and vegetation (Čeirāns, 2007; Strijbosch, 1988; Vos et al., 2007) as well as a preference of *V*. *berus* to habitats proximal to the forest edge as already shown for several snake species (Carfagno et al., 2006; Nöllert, 2004; Scali et al., 2008). More surprising is the close association we found between rocky formations and *V*. *berus*, which is considered one of the least dependent on this resource among the European vipers (Mebert et al., 2015). Despite our predictions, we found that it is the availability of microhabitats within ski‐runs, hence their management, that determines their suitability as habitats for reptiles and amphibians, and we believe that certain ski‐runs, given the persistence of microhabitat patches, might actually be beneficial for reptiles and amphibians, similarly to what power‐line clearings do for oviparous reptiles in mountain ecosystems (Shine et al., 2002). A preliminary survey conducted along two plots in 2018 showed in fact that none of the three species was found inside the surrounding forests (data not shown), probably because of the too low temperature, which make it an unsuitable habitat for ectothermic species (Huang et al., 2014). The increased solar radiation of the ski‐runs might therefore allow these species to move between patches of forests and to colonise areas that would otherwise be inaccessible.

More complex is to disentangle the effects of climate on the year‐to‐year occupancy changes of the species. While most studies agree on the importance of humid springs for favouring lizards' gestation, survival, and recruitment (Brusch IV et al., 2020; Dezetter et al., 2021; Le Galliard et al., 2010), it has been suggested that this does not apply in mountain environments (Marquis et al., 2008). Our results support this latter hypothesis, and expand it also to *Vipera berus*, which experienced the same reduction in occupancy following rainy springs (Figure 4). However, for both reptile species, spring precipitation was only one of the factors influencing disappearance dynamics and changes in the density of microhabitats, such as bushes, logs and ground vegetation, were relevant as well. On the other hand, post‐metamorphosis rainfall was the only parameter relevant for *R*. *temporaria* disappearance, with a strong correlation between rain and occupancy in the following year (Figure 4). Due to the very short time between metamorphosis and hibernation that juvenile amphibians experience in alpine environments (Miaud et al., 1999; Ryser, 1996), maximising the accumulation of fat storage is crucial to surviving winter (Scott et al., 2007); however, activity rates is substantially shortened if conditions are too dry (Bredeweg et al., 2019). Finally, winter temperatures appear to be more marginal, confirming that pre‐ or post‐hibernation conditions are more determinant in causing amphibian mortality than overwintering ones (Bauwens, 1981; Bauwens & Claus, 2019).

Effective and sustainable management of ski‐runs is becoming a pressing aspect of the winter tourism industry (Rivera & de Leon, 2004), and more and more resort owners try to implement specific regulations and effective practices in order to ensure a successful environmental restoration (Pintaldi et al., 2017). Because of our study species' adaptability and tolerance, a mild set of adaptations, such as the preservation of natural microhabitats along the ski‐runs, could be enough to ensure their survivability in most ski resorts of the Alps. The removal of woody debris, boulders and vegetation is the primary limit in reptile and amphibian survival since it deprives them of sites for basking, shelters from predators as well as from extreme temperatures. Therefore, in the specific case of herpetofauna conservation, buffering the edges of ski‐runs with native vegetation, or building piles of boulders, logs and similar debris removed during the construction to the edges could satisfy the basic requirements for the species and provide enough shelters, basking sites and hunting grounds (Sato, Schroder, Green, Michael, et al., 2014; Sato, Wood, Schroeder, Green, et al., 2014). Moreover, in order to make the centre of the ski run more suitable and to preserve the arthropod prey stock within it, minimising machine‐grading would help in preserving the original vegetation cover (Negro et al., 2009), while the use of site‐specific seed mixtures for revegetation produces a healthier and more diverse plant community that can more easily survive and function (Krautzer et al., 2006; Pintaldi et al., 2017). Although this is not proven in our cases, other studies also showed how reptile species tend to avoid patches of non‐native vegetation and therefore the use of local plants should be favoured (Sato, Wood, Schroeder, et al., 2013).

However, these recommendations do not apply exclusively to the management of ski‐runs. Grazing for instance is one of the main forms of land use in alpine areas (Zhao et al., 2018), and its excess has resulted in several forms of environmental deterioration, such as soil erosion (Torresani et al., 2019), species depletion (Negro et al., 2011) and desertification (Cao et al., 2004). In the light of our results, particularly relevant is the effect that overgrazing can have on vegetation cover and plant height (Kruess & Tscharntke, 2002). While in fact overgrazing does not seem to affect the number of plant species in alpine regions, it certainly reduces their biomass and height (Zhang et al., 2015), hence eliminating available shelters for several animal species, including amphibians and reptiles.

Nevertheless, we acknowledge several limitations to our study. Above all, the generalist ecology of our focal species is not representative of the whole alpine herpetofauna. For instance, a specialised predator, such as the meadow viper (*Vipera ursinii*), whose main prey species are severely affected by ski‐runs management (Negro et al., 2010), might be more threatened than a species like *V*. *berus*, which feeds on a broader range of prey. Moreover, Passo Rolle is a quite small ski resort, and this might reduce its effects on biodiversity compared to larger and more crowded resorts, where the density of ski‐runs and connected infrastructure is higher. Finally, it is also possible that the survey design, which preferred morning surveys, biased the results, particularly regarding plots that received solar light later in the day, although the insignificance of the orientation covariate in the occupancy model seems to reject this limitation.

## 5 CONCLUSION

Our results indicate the combined role of micro‐ and macro‐scale factors in determining the presence and permanence of reptile and amphibian species in high‐altitude environments. Regarding ski‐runs specifically, these habitats are not necessarily bad or good for these species. On the one hand, the oversimplification of the habitat following an excessive maintenance would deprive such species of the microhabitats necessary to survive. On the other hand, these habitats might offer a better thermal environment than the surrounding forests, hence working as corridor for ectothermic species and potential refuges counterbalancing the expansion of woods. We believe that a sustainable management is the best strategy to secure both the survivability of these species and the economic income that is fundamental for many alpine communities.

## AUTHOR CONTRIBUTIONS

**Michele Chiacchio:** Conceptualization (equal); formal analysis (lead); investigation (equal); methodology (equal); writing – original draft (lead). **Dennis Rödder:** Formal analysis (supporting); investigation (equal); methodology (equal); supervision (equal); writing – review and editing (equal). **Klaus Henle:** Conceptualization (equal); investigation (equal); methodology (equal); supervision (equal); writing – review and editing (equal). **Annegret Grimm‐Seyfarth:** Conceptualization (equal); formal analysis (supporting); investigation (equal); methodology (equal); supervision (equal); writing – review and editing (equal).

## CONFLICT OF INTEREST STATEMENT

No actual or potential conflict of interest to declare.

## Data Availability

Data deposited in the Dryad Digital Repository: https://datadryad.org/stash/share/phTumE9zdy0NsgrEU15M9AKZAjg3lgKoXCtBkFd3CTs (Chiacchio et al., 2024).
